# The reliability and quality of short videos as health information of guidance for lymphedema: a cross-sectional study

**DOI:** 10.3389/fpubh.2024.1472583

**Published:** 2025-01-03

**Authors:** Xuchuan Zhou, Gejia Ma, Xuefeng Su, Xinyi Li, Wenfei Wang, Linxi Xia, Chen Yang, Bin Liu

**Affiliations:** ^1^Department of Burn, Plastic and Cosmetic Surgery, Xi’an Central Hospital, Xi’an Jiaotong University, Xi’an, China; ^2^School of Medicine, Yan’an University, Yan’an, China

**Keywords:** lymphedema, information quality, social media, short videos, TikTok

## Abstract

**Background:**

The prevalence of lymphedema is rising, necessitating accurate diagnostic and treatment information for affected patients. Short video-sharing platforms facilitate access to such information but require validation regarding the reliability and quality of the content presented. This study aimed to assess the reliability and quality of lymphedema-related information on Chinese short video-sharing platforms.

**Methods:**

We collected 111 video samples addressing the diagnosis and treatment of lymphedema from four platforms: TikTok, Bilibili, WeChat, and Microblog. Two independent surgeons evaluated each video for content comprehensiveness, quality (using the Global Quality Score), and reliability (using the modified DISCERN tool). The videos from different sources were subsequently compared and analyzed.

**Results:**

Out of 111 videos analyzed, 66 (59.46%) were uploaded by medical professionals, including breast surgeons, vascular surgeons, plastic surgeons, physical therapists, and gynecologists, while 45 (40.54%) were shared by non-medical professionals such as science bloggers, medical institutions, and lymphedema patients. Patient-uploaded videos received the highest engagement, with median likes of 2,257 (IQR: 246.25–10998.25) and favorites of 399 (IQR: 94.5–1794.75). 13 videos (11.71%) contained inaccuracies. Medical professionals’ videos generally showed higher content comprehensiveness, particularly those by plastic surgeons, compared to non-medical professionals. The GQS and modified DISCERN tool were used to assess video quality and reliability respectively, with medical professionals scoring higher on both metrics (*z* = 3.127, *p* = 0.002; *z* = 2.010, *p* = 0.044). The quality and reliability of recommendations provided by plastic surgeons surpassed that of other medical professionals (*χ*^2^ = 16.196, *p* = 0.003; *χ*^2^ = 9.700, *p* = 0.046). No significant differences in video quality and reliability were found among the three categories of non-medical professionals (*χ*^2^ = 3.491, *p* = 0.175; *χ*^2^ = 2.098, *p* = 0.350).

**Conclusion:**

Our study shows that lymphedema-related videos on short video platforms vary widely in quality. Videos by medical professionals are generally more accurate and of higher quality than those by non-professionals. However, patient-uploaded videos often get more engagement due to their relatability. To ensure public access to reliable information, establishing basic standards for this content is essential.

## Introduction

Lymphedema is a chronic condition characterized by the buildup of lymphatic fluid in tissues due to compromised lymphatic circulation (1). This condition can lead to swelling, discomfort, reduced mobility, and skin changes, typically affecting the arms, legs, genitals or other areas of the body (2–6). Prolonged lymphatic stasis can lead to inflammatory responses in the affected limb, adipose tissue hyperplasia, and tissue fibrosis (7, 8). Without timely intervention, the affected area may develop permanent and severe swelling, resulting in disability and causing significant negative impacts on the patient’s physical, social, and psychological well-being (6, 9, 10).

Enhanced understanding of lymphedema management principles is critical for improving patient outcomes. Health education for chronic disease patients aims to provide them with the skills needed to manage their condition effectively (11). For lymphedema patients, proper education can promote early detection, diagnosis, and treatment, while also reducing postoperative complications such as cellulitis and erysipelas (12).

Due to the disparity in economic and medical development across regions in China, particularly in remote areas, patients with lymphedema often struggle to obtain accurate diagnoses and appropriate treatments. Finding accessible, easy-to-understand information about their condition remains challenging. Over the past 30 years, advancements on the internet have improved access to medical consultations and health education for patients (13). In the last 5 years, the emergence of 5G technology and the widespread adoption of smartphones in China have prompted more consumers to utilize social media platforms, such as TikTok, Bilibili, WeChat, and Microblog, for health education and to select medical resources (11, 14, 15).

Consequently, short video platforms in China are evolving as vital sources of health knowledge and education for patients and as new avenues for physicians to connect with them. Evidence increasingly supports the effectiveness of video education; for instance, a randomized controlled trial found that video-based education significantly enhanced disease knowledge among atopic dermatitis patients compared to written materials (16–20). Studies on the cost-effectiveness of digital health interventions, including network-based consultations, further highlight the potential for these methods to reduce healthcare expenses compared to traditional face-to-face consultations (21, 22).

These platforms offer unique advantages in disseminating health information. Visual content, particularly video, is often easier to absorb and remember than textual information (11, 23, 24). It may help patients with lymphedema assess the severity of their condition and pursue standardized diagnostics and treatment. Thus, utilizing social media for standardized communication in lymphedema management is essential.

However, social media also presents limitations in the dissemination of health information. The prevalence of incorrect health information increases the risk that patients may make misguided health decisions based on unreliable sources (25). Patients should learn to differentiate between high-quality and low-quality information (26, 27). Consequently, evaluating the quality of health information on social media is crucial.

TikTok, Bilibili, WeChat, and Microblog have attracted over 1 billion users in China due to their ease of use and accessible resources. On these platforms, patients can find numerous health-related videos about lymphedema by simply entering relevant keywords in the search bar. However, the quality of lymphedema-related videos has not yet been thoroughly evaluated. This study aimed to assess the information quality of lymphedema-related videos on TikTok, Bilibili, WeChat, and Microblog.

## Methods

### Search strategy and data processing

Video retrieval occurred between December 1, 2023, and March 1, 2024. The search keywords included 淋巴水肿 (“lymphedema”), 上肢水肿 (“upper limb edema”), and 下肢水肿 (“lower limb edema”). All collected videos were sourced from TikTok, Bilibili, WeChat, and Microblog, the 4 most popular Chinese social media platforms. Videos were excluded from the analysis if they were copied, lacked proper authorship attribution, or were created for commercial purposes (e.g., selling compression garments, elastic bandages, or skincare products). Videos consisting of multiple parts were considered as a single entity. Basic information was extracted from each video, including the uploader’s identity, video length, number of saves, shares, and likes. All the data was recorded in Excel (Microsoft Corporation).

### Evaluating methodologies

The content, reliability, and quality of the videos were evaluated. First, based on the guidelines issued by the International Society of Lymphology (ISL) in 2020, combined with the disease coding model which was the standard for characterizing misleading information, we scored the quality of lymphedema-related videos in 6 categories: definition, symptoms, related risk factors, how to evaluate, treatment/management and outcomes of lymphedema (8, 13, 25, 28). The scoring criteria were as follows: 0 points (no content), 0.5 points (minimal content), 1 point (some content), 1.5 points (most content), and 2 points (extensive content).

The study employed a modified DISCERN tool to evaluate the overall reliability of the video content. DISCERN is a widely recognized instrument used in research to assist consumers and healthcare providers in assessing the quality of health information (11, 29). As indicated in Appendix 1, DISCERN evaluates video content quality through 5 criteria: clarity, relevance, traceability, robustness, and fairness. Each question receives a score of 1 point for a “yes” response and 0 points for a “no” response, resulting in a possible score ranging from 0 to 5 points.

As indicated in Appendix 2, we utilized the Global Quality Score (GQS) to evaluate the quality of the videos. The GQS is commonly employed to assess the quality of health information on online video platforms, with videos rated on a 5-point scale, where 1 indicates poor quality and 5 signifies excellent quality (11, 15, 30, 31).

### Evaluation procedure

Before the search, the two raters (XY L and YC) responsible for evaluating video quality had not viewed any lymphedema-related videos. To eliminate potential biases from personal recommendation algorithms, we deleted all historical records and settings from the smartphone, applied for hosting, and logged into a new account on each video platform.

**Figure 1:**
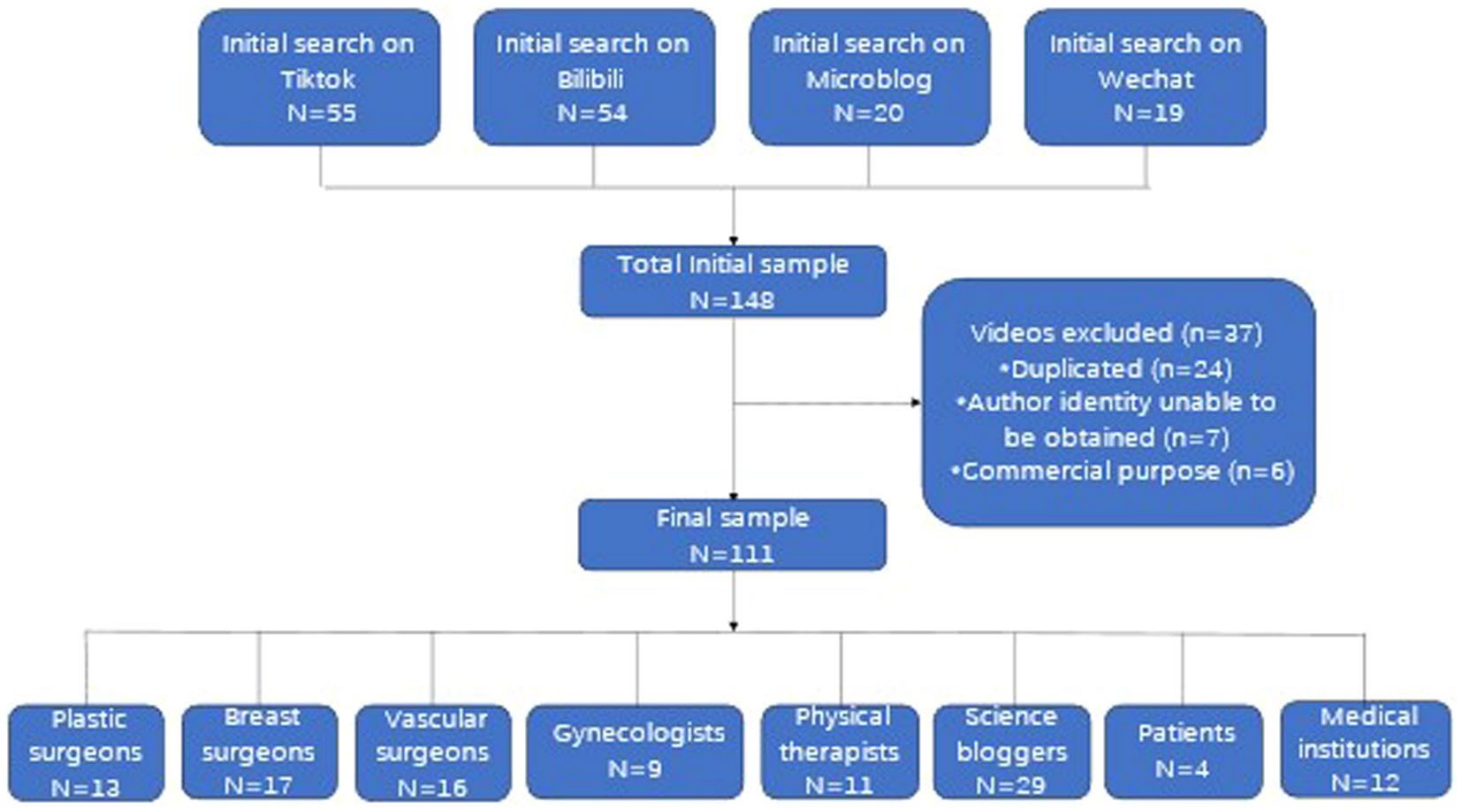
Video screening procedure.

Based on the inclusion and exclusion criteria, 111 lymphedema-related videos were selected for further analysis (Figure 1). These videos were categorized into two groups according to the uploader’s identity: medical professionals and non-medical professionals. Within the medical professional group, video creators were further classified into five categories: plastic surgeons, breast surgeons, vascular surgeons, gynecologists, and physical therapists. For videos uploaded by non-medical professionals, creators were categorized into three groups: science bloggers, medical institutions, and lymphedema patients.

The raters assessed each video based on several criteria. First, they recorded fundamental video information that was mentioned in search strategy and data processing, including video length, number of favorites, shares, likes, comments and the uploader’s identity. Second, the raters independently evaluated the content, reliability, and quality of each video using the disease coding mode, DISCERN and GQS. Prior to scoring, the raters reviewed the official scoring instructions for DISCERN and GQS. Each video was assessed by both raters, followed by discussions to resolve discrepancies.

### Statistical analysis

The inter-rater reliability was determined by calculating the Cohen *κ* coefficient. The reliability of the 6 items related to the disease coding model ranged from 0.827 to 0.932. The inter-rater reliability of DISCERN and GQS was 0.851 and 0.826, respectively. These results indicate satisfactory inter-rater reliability.

SPSS 24.0 statistical software was used to analyze the data. The normally distributed data was expressed as mean and standard deviation (SD), whereas the non-normally distributed measurements were expressed as median and interquartile range (IQR). Comparisons between 2 groups were performed using non-parametric Mann–Whitney tests, while comparisons among 3 or more groups were made with the Kruskal–Wallis *H* test. The count data were expressed as a rate (%) and analyzed by chi-square (*χ*^2^) test. The correlations between different datasets were analyzed using Spearman correlation analysis. Statistical significance was set at *p* < 0.05. R software (version 4.3.0) and Origin 2021 were used for statistical analysis and data visualization.

## Results

Of the 111 included videos, 66 (59.46%) were uploaded by medical professionals, while 45 (40.54%) were shared by non-medical professionals. Among medical professionals, the largest contributors were breast surgeons (*n* = 17, 15.32%), followed by vascular surgeons (*n* = 16, 14.41%), plastic surgeons (*n* = 13, 11.71%), physical therapists (*n* = 11, 9.91%), and gynecologists (*n* = 9, 8.11%). In contrast, most videos from non-medical professionals were created by science bloggers (*n* = 29, 26.13%), followed by medical institutions (*n* = 12, 10.81%) and lymphedema patients (*n* = 4, 3.60%). The 111 lymphedema-related videos analyzed collectively garnered 151,730 “likes,” 39,599 “favorites,” and 25,389 “shares.” Videos uploaded by patients received the highest levels of engagement, with a median number of likes at 2,257 (IQR: 246.25–10998.25) and favorites at 399 (IQR: 94.5–1794.75). At the same time, the 111 lymphedema related videos analyzed received a total of 12,642 comments, of which 1,517 (12.00%) were comments about consulting and discussing treatment plan, and only 121 (7.98%) comments received valid responses. The characteristics of the different video groups are detailed in Table 1.

**Table 1:** Characteristics of the videos across sources.

Source (description)	Videos, *n* (%)	Length of video (seconds), median (IQR)	Likes, median (IQR)	Favorites, median (IQR)	Times shared, median (IQR)
Medical professionals					
Plastic surgeons	13(11.71)	975(161.5–2,206)	698(204–3459.5)	190(21–1,124)	197(39–660)
Breast surgeons	17(15.32)	242(51.5–1111.5)	18(2.5–117.5)	11(2.5–215)	9(1.5–242.5)
Vascular surgeons	16(14.41)	148(54.5–1074.5)	339.5(6.5–907)	47.5(2–231.5)	70(4.25–310.5)
Gynecologists	9(8.11)	164(113.5–399.5)	501(153.5–2112.5)	378(40.5–849.5)	303(40–855)
Physical therapists	11(9.91)	398(240–1,299)	89(27–781)	67(8–230)	28(5–201)
Non–medical professionals					
Science bloggers	29(26.13)	249(52.5–521.5)	12(3–69.5)	30(0.5–95.5)	12(1–39.5)
Patients	4(3.60)	932.5(303.75–2802.50)	2,257(246.25–10998.25)	399(94.5–1794.75)	18.5(5.25–28.75)
Medical institutions	12(10.81)	120.5(51.75–586.25)	2.5(1–140.25)	1(0–23.75)	1.5(0.25–64)

Spearman correlation analysis was conducted to examine the relationships between video length and the number of likes, favorites, and shares. The analysis revealed a slight positive correlation between video length and the number of favorites (*r* = 0.385, *p* = 0.001). Additionally, weak positive correlations were observed between video length and the number of likes (*r* = 0.293, *p* = 0.002) as well as shares (*r* = 0.233, *p* = 0.014).

### Video content

Among the 111 videos, 40 focused on upper limb lymphedema, 25 on lower limb lymphedema, and 46 addressed both upper and lower limb lymphedema. A total of 13 videos (11.71%) contained content with varying degrees of inaccuracies. The disease coding model was applied to assess the comprehensiveness of the video content. As shown in Table 2, few videos provided standardized and comprehensive health education regarding lymphedema. Only 6.31% included information on outcomes (classified as “most” or “extensive” content).

**Table 2:** Completeness of video content.

Video content	Definition, *n*(%)	Symptoms, *n*(%)	Risk factors, *n*(%)	Evaluation, *n*(%)	Management, *n*(%)	Outcomes, *n*(%)
No content (0 points)	15(13.51)	26(23.42)	25(22.52)	22(19.82)	19(17.12)	16(14.41)
Few content (0.5 points)	47(42.34)	35(31.53)	45(40.54)	51(45.95)	43(38.74)	64(57.66)
Some content (1 point)	34(30.63)	38(34.23)	25(22.52)	23(20.72)	35(31.53)	24(21.62)
Most content (1.5 points)	14(12.61)	12(10.81)	16(14.41)	14(12.61)	14(12.61)	6(5.41)
Extensive content (2 points)	1(0.90)	0(0)	0(0)	1(0.90)	0(0)	1(0.90)

**Figure 2:**
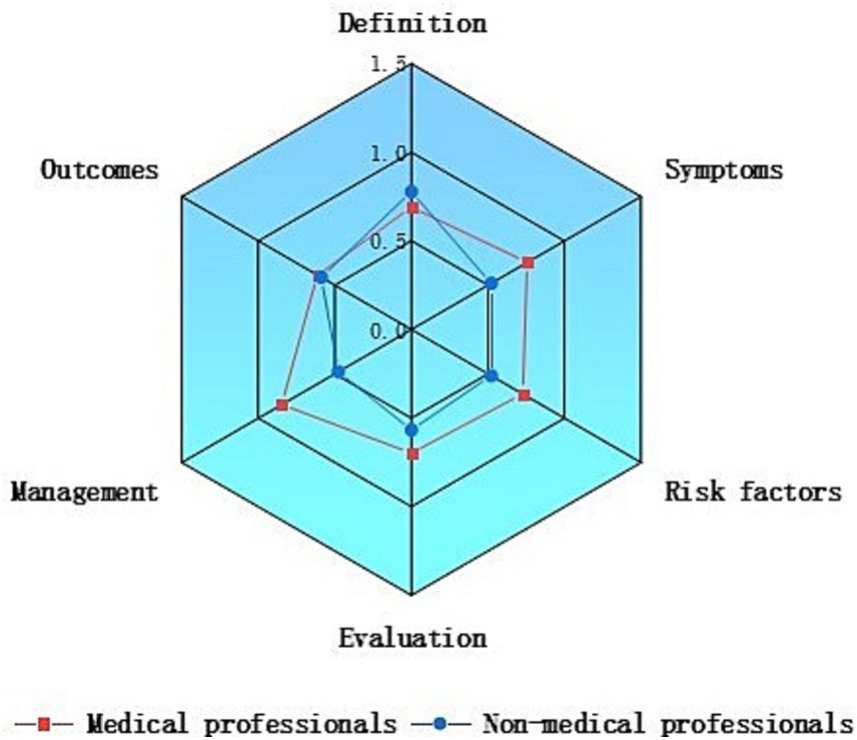
Comparison of content comprehensiveness between medical professionals and non-medical professionals.

As shown in Table 2, most video content focused on the definition, symptoms, and treatment/management of lymphedema, accounting for 44.14, 45.05, and 44.14% (classified as “some”, “most”, or “extensive” content), respectively. Additionally, 63.06% of the videos did not mention or rarely mentioned risk factors for lymphedema (classified as “no” or “few” content). We then compared content comprehensiveness across different video sources. As illustrated in Figure 2, aside from definitions, videos created by medical professionals had higher content coverage in the other five categories, with an overall content score superior to that of non-medical professionals.

**Figure 3:**
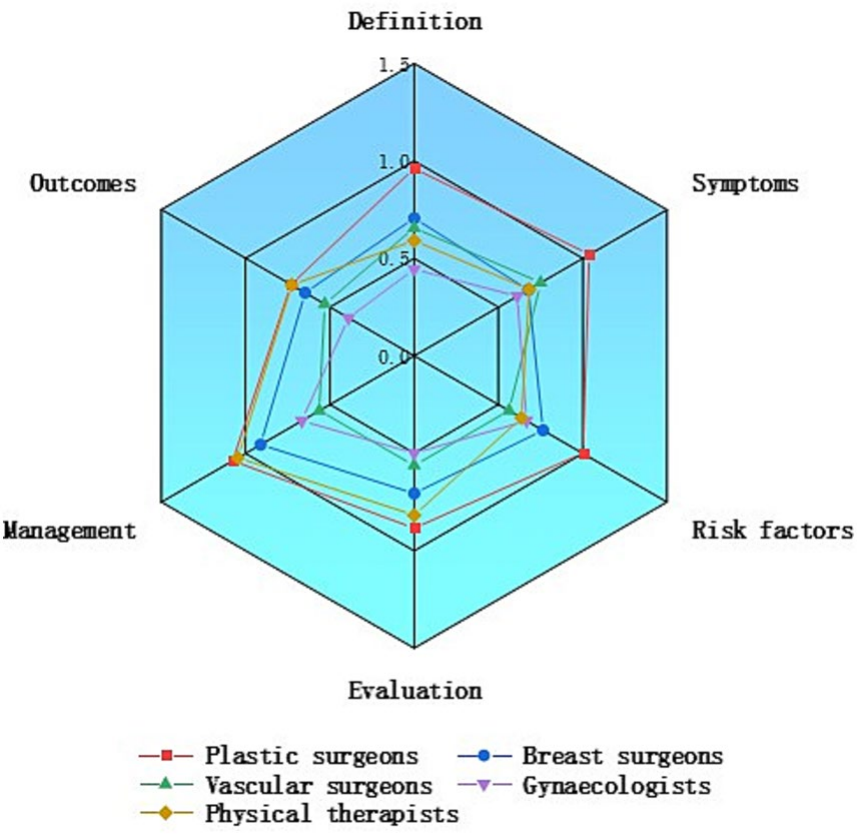
Comparison of content comprehensiveness between different medical professionals.

**Figure 4:**
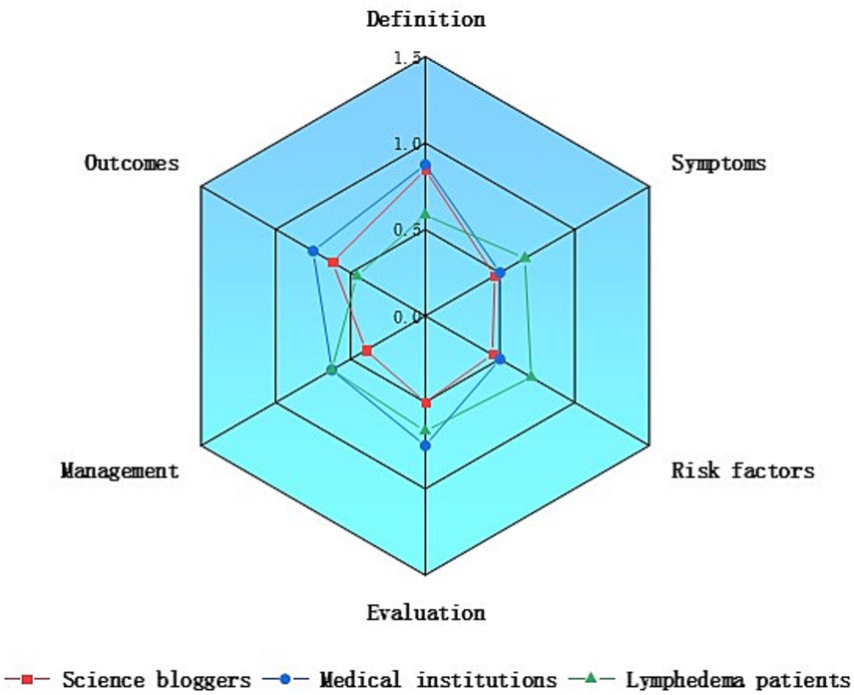
Comparison of content comprehensiveness between different non-medical professionals.

Further evaluation of the two groups’ content revealed that, among medical professionals, plastic surgeons provided the most comprehensive video content, offering more extensive health education and treatment options for patients with lymphedema (Figure 3). In the non-medical professional group, videos from patients contained more information on lymphedema symptoms and risk factors, whereas videos from medical institutions and science bloggers placed greater emphasis on definitions and outcomes (Figure 4).

### Information quality and reliability

**Figure 5:**
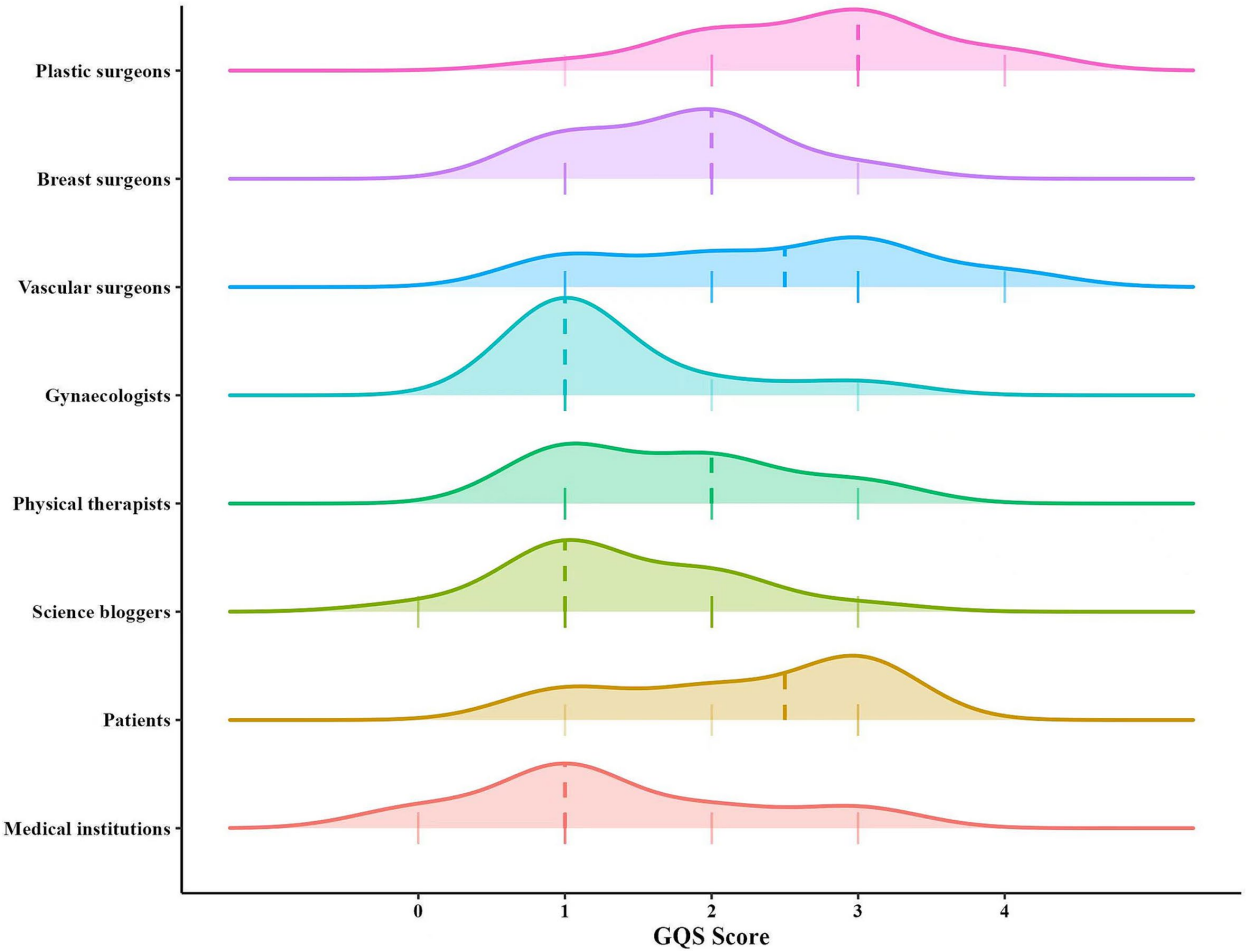
Comparisons of Global Quality Score among different sources.

We first use GQS to assess the overall quality of each video. As shown in Table 3, the average GQS score for all videos was 1.79 (SD 0.94). Videos created by medical professionals scored higher on the GQS than those from non-medical professionals (*z* = 3.127, *p* = 0.002). Further analysis indicated that videos by plastic surgeons had higher quality scores than those from breast surgeons, vascular surgeons, gynecologists, and physical therapists (*χ*^2^ = 16.196, *p* = 0.003) (Figure 5). There were no significant differences in GQS scores among the three non-medical professional groups (*χ*^2^ = 3.491, *p* = 0.175) (Figure 5).

**Table 3:** GQS and DISCERN scores of the videos across sources.

Source (Description)	GQS scores, median (IQR)	GQS scores, mean (SD)	DISCERN scores, median (IQR)	DISCERN scores, mean (SD)
Medical professionals				
Plastic surgeons	3(2–3)	2.69(0.85)	3(1.5–3)	2.46(1.05)
Breast surgeons	2(1–2)	1.76(0.66)	1(1–2)	1.65(0.79)
Vascular surgeons	2.5(1.25–3)	2.36(1.02)	1(1–2)	1.44(1.09)
Gynecologists	1(1–1.5)	1.33(0.71)	1(1–2)	1.44(1.24)
Physical therapists	2(1–2)	1.73(0.79)	2(1–2)	1.64(0.67)
Non–medical professionals				
Science bloggers	1(1–2)	1.38(0.73)	1(1–2)	1.31(0.66)
Patients	2.5(1.25–3)	2.25(0.96)	2(1–3)	2.00(1.15)
Medical institutions	1(1–2)	1.33(0.98)	1(1–1.75)	1.25(0.97)

**Figure 6:**
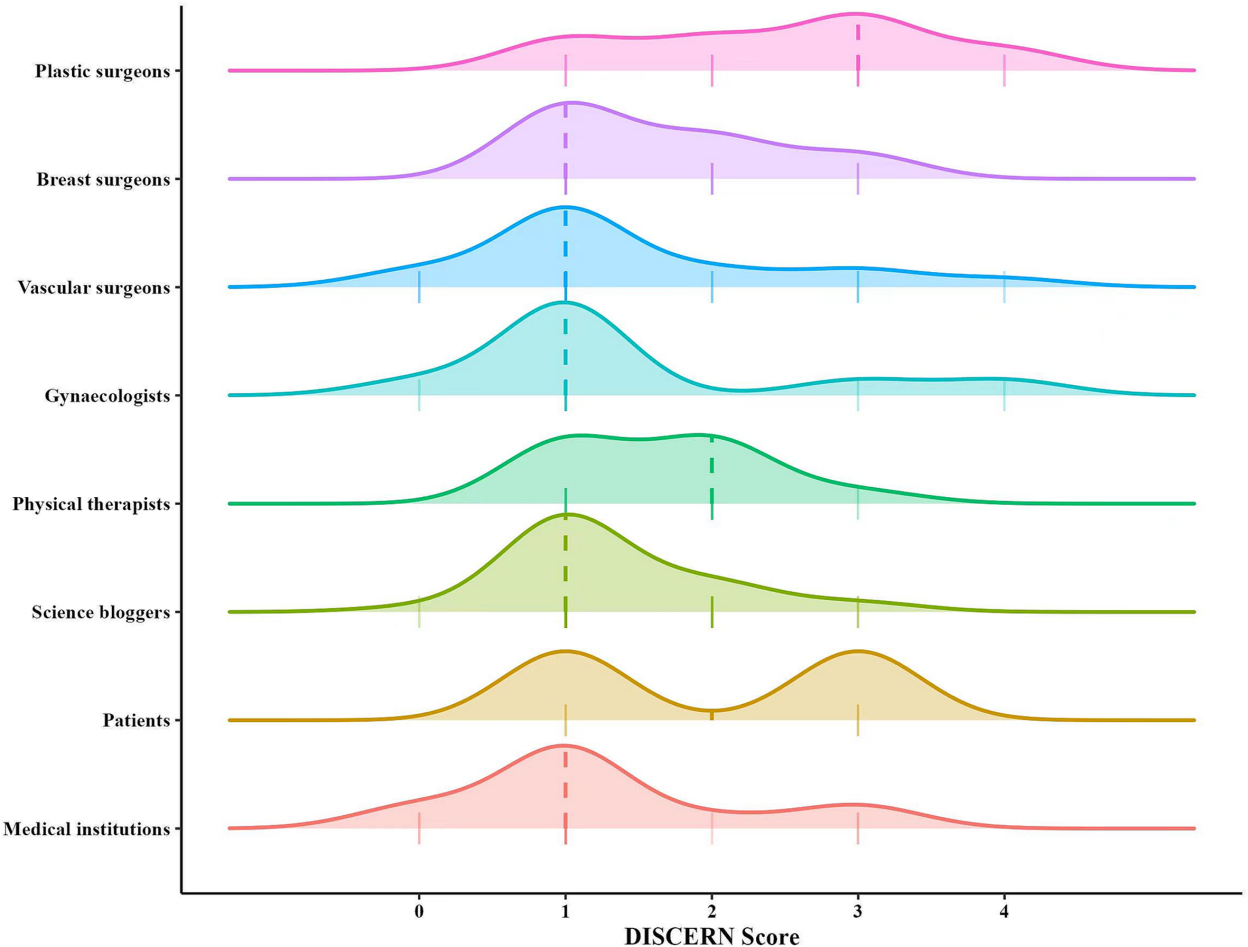
Comparisons of DISCERN Score among different sources.

The DISCERN was used to assess video reliability, with results similar to those of the GQS. The average DISCERN score across all videos was 1.58 (SD 0.95) (Table 3). Consistent with the GQS findings, reliability scores for videos by medical professionals were higher than those by non-medical professionals (*z* = 2.010, *p* = 0.044). Additional analysis revealed that videos from plastic surgeons also had higher DISCERN scores than those from breast surgeons, vascular surgeons, gynecologists, and physical therapists (*χ*^2^ = 9.700, *p* = 0.046) (Figure 6). As with GQS, there was no significant difference in DISCERN scores among the three non-medical professional groups (*χ*^2^ = 2.098, *p* = 0.350) (Figure 6).

Furthermore, Spearman correlation analysis was also performed to assess the relationship between video length and scores for content comprehensiveness, GQS, and DISCERN. The results indicated that content comprehensiveness, GQS, and DISCERN scores were moderately positively correlated with video length (*r* = 0.579, *p* = 0.001; *r* = 0.570, *p* = 0.001; *r* = 0.553, *p* = 0.001).

## Discussion

Recent studies have indicated that patients with chronic diseases increasingly rely on social media for information regarding disease diagnosis and treatment (32). We evaluated the comprehensiveness, quality, and reliability of lymphedema videos across several platforms, including TikTok, Bilibili, WeChat, and Microblog. Our findings demonstrate that short video-sharing platforms serve as effective channels for disseminating lymphedema-related information.

This study shows that the length of lymphedema video has a certain correlation with the number of favorites, likes, shares, content comprehensiveness, quality and reliability. These findings have practical implications for lymphedema -related content on platforms. While shorter videos may capture audience attention quickly, their ability to convey comprehensive and high-quality information appears limited. Conversely, longer videos contribute more meaningfully to educational and informational goals. This creates a trade-off for content creators and platforms between maximizing engagement and ensuring content quality.

Lymphedema, a chronic and often debilitating condition, necessitates accurate and evidence-based interventions to optimize patient outcomes (33–35). Our comprehensive review of lymphedema related videos revealed incorrect treatment recommendations in terms of the accuracy and reliability of the information provided by some videos.

For example, 3 uploaders incorrectly asserted that patients with early-stage lymphedema do not require compression therapy following lymphatic venous anastomosis (LVA). This statement is not only inconsistent with ISL guidelines but also fails to consider the role of compression in supporting the surgical outcomes of LVA by promoting lymphatic flow and reducing edema (8). Compression therapy remains a cornerstone of early lymphedema management, even in surgical contexts (36, 37).

Moreover, 4 videos demonstrated non-standardized bandaging techniques, which deviated significantly from established best practices. Correct bandaging technique is integral to the success of decongestive therapy. Improper application can lead to suboptimal results or even exacerbate lymphedema by causing uneven pressure distribution or discomfort for patients. Additionally, 5 videos showcased manual lymphatic drainage (MLD) techniques that were not standardized.

Liposuction, widely utilized as a surgical intervention for advanced lymphedema, has been shown to alleviate swelling, reduce the frequency of cellulitis, and mitigate fibrosis in the affected limb (8, 36, 38–40). However, our review found limited video content discussing or recommending liposuction, possibly because only plastic surgeons in China are licensed to perform this procedure. In the future, increasing awareness and accessibility of liposuction as a treatment for lymphedema should be a priority.

The analysis of the 111 lymphedema-related videos reveals a concerning lack of patient interaction and engagement within the comments section. While the videos collectively garnered 12,642 comments, only 1,517 (12.00%) were directly related to the condition and its treatment, suggesting that the majority of viewer interactions may center on unrelated discussions rather than substantive inquiries about lymphedema. Only 121 (7.98%) of these condition-specific comments received valid responses, indicating a significant gap in two-way communication between content creators and viewers.

Our findings indicate that few videos adequately cover all aspects of lymphedema science or provide appropriate, credible recommendations, particularly regarding disease management and treatment. Only 12.6% of the videos presented a comprehensive, standardized treatment plan for lymphedema. The findings indicate a need for improved quality in lymphedema-related videos. There is significant variability in the quality of videos uploaded by different individuals. With the rising popularity of video-sharing platforms, it is essential to establish basic standards for lymphedema-related video content.

The potential for digital health in chronic disease management, including lymphedema, is substantial. However, further studies with diverse patient populations are needed to fully understand its impact, alongside policy measures that encourage patient engagement in digital health to improve healthcare efficiency. While the Chinese government has issued regulatory guidelines on health information dissemination via new media, no international standards currently address quality assurance for health education videos.

Short video platforms could benefit from establishing dedicated “health” sections where only verified or professionally reviewed videos are permitted. Such sections could serve as centralized hubs for evidence-based content, addressing the growing concerns regarding misinformation and its potential harm to public health. However, the implementation of this concept requires careful planning to balance accessibility, accuracy, and inclusivity. First, only verified medical professionals, licensed institutions, and reputable patients or bloggers should be authorized to contribute content. Verification labels could be used to clearly identify these creators, providing viewers with an indicator of credibility. Second, videos submitted to the health section should undergo a peer review or fact-checking process conducted by a panel of qualified healthcare experts. This ensures the dissemination of scientifically accurate and up-to-date information. Third, to maintain user engagement, these sections could integrate interactive features such as Question and Answer sessions with professionals, live streams, or mechanisms for user feedback to address specific health concerns.

The findings indicate that videos uploaded by patients garnered the highest engagement in terms of likes and collections, with median values of 2,257 (IQR: 246.25–10998.25) likes and 399 (IQR: 94.5–1794.75) favorites. This trend highlights the appeal of patient-generated content, which often resonates with audiences due to its relatability, emotional connection, and authenticity. Unlike content created by medical professionals, which typically emphasizes accuracy and comprehensiveness, patient-uploaded videos may focus more on personal experiences and practical insights, making them more engaging and shareable among viewers.

However, very few people with lymphedema post videos about their own experiences and views on treatment. Many patients may not feel comfortable sharing their personal health experiences on public platforms, fearing stigma, judgment, or a lack of relevance to others. This hesitancy may be heightened by the often visible physical symptoms of lymphedema, which can affect self-image and willingness to appear on camera. Patients also might feel that their experiences are too niche or lack the authority to contribute meaningfully to a broader audience.

Incorporating contributions from non-medical professionals within a dedicated “health” section, particularly for lymphedema-related content, offers unique value while broadening the diversity of available information. Although non-medical professionals lack formal clinical training, their lived experiences, personal insights, and practical advice can complement professional knowledge, making the content more relatable and accessible to patients and caregivers. Patient-led content, in particular, can foster a sense of community and reduce the isolation experienced by individuals living with lymphedema.

While video content can simplify complex information, barriers to health education remain, especially for patients unfamiliar with medical terminology. To address this, medical professionals should strive to make their content more comprehensible, while non-professionals must ensure the accuracy of their information. Standardized health education videos should be scientifically valid and accessible to a general audience. A hybrid approach that combines the credibility of medical professionals with the relatability of non-professionals could maximize the reach and impact of lymphedema-related video content, thereby improving public understanding and patient outcomes. Given the variability in video quality, platforms must curate and prioritize high-quality, comprehensive educational videos to better guide patients and enhance the dissemination of accurate health information.

### Limitation

This study has several limitations. First, selection bias is present, as it focuses only on lymphedema-related videos from a limited number of Chinese platforms (TikTok, Bilibili, WeChat, and Microblog), which may not capture the global context. Second, the evaluation relied on researchers’ subjective interpretations, potentially introducing subjective bias and affecting replicability. Additionally, time bias is a factor, given the dynamic nature of social media; the videos analyzed represent only a specific point in time, which may impact the relevance of our findings. Lastly, the study did not evaluate the long-term effects of video-based health information on patient outcomes. Future research should include a broader range of platforms and consider longitudinal studies to assess long-term effectiveness.

## Conclusion

Our findings indicate that the quality of lymphedema-related videos on short video platforms remains suboptimal, with notable variability in comprehensiveness, reliability and quality depending on the uploader. Videos created by medical professionals generally outperform those produced by non-professionals in terms of factual accuracy and content quality. However, videos uploaded by non-professionals, particularly patients, often garner more likes, favorites, and shares, likely due to their relatability and emotional appeal. Given the rising popularity of video-sharing platforms, establishing basic standards for lymphedema-related video content should be prioritized to ensure accurate and reliable information is accessible to the public.

## Data Availability

The original contributions presented in the study are included in the article/Supplementary material, further inquiries can be directed to the corresponding author.
